# *APC* hypermethylation for early diagnosis of colorectal cancer: a meta-analysis and literature review

**DOI:** 10.18632/oncotarget.17576

**Published:** 2017-05-02

**Authors:** Tie-Jun Liang, Hong-Xu Wang, Yan-Yan Zheng, Ying-Qing Cao, Xiaoyu Wu, Xin Zhou, Shu-Xiao Dong

**Affiliations:** ^1^ Department of Digestive Disease, Shandong Provincial Hospital Affiliated to Shandong University, Jinan, Shandong, China; ^2^ Department of General Surgery, Jiyang People's Hospital, Jiyang, Shandong, China; ^3^ Department of Medical Imaging, Jiyang People's Hospital, Jiyang, Shandong, China; ^4^ Department of Anus & Intestine Surgery, Taian City Central Hospital, Taian, Shandong, China; ^5^ Department of Surgical Oncology, The Affiliated Hospital of Nanjing Medical University, Nanjing, Jiangsu, China; ^6^ Department of General Surgery, Jiangsu Cancer Hospital, The Affiliated Cancer Hospital of Nanjing Medical University, Nanjing, Jiangsu, China; ^7^ Department of Gastrointestinal Surgery, Linyi People's Hospital, Linyi, Shandong, China

**Keywords:** adenomatous polyposis coli, APC, methylation, biomarker, adenoma

## Abstract

*Adenomatous polyposis coli* (*APC*) promoter hypermethylation has been frequently observed in colorectal cancer (CRC). The association between *APC* promoter methylation and clinicopathological significance in CRC is under investigation. We performed a meta-analysis to quantitatively evaluate the significance of *APC* methylation in CRC. The study included a total of 24 articles and 2025 CRC patients. The frequency of *APC* promoter hypermethylation was significantly higher in colorectal adenoma than in normal colorectal tissue, OR was 5.76, 95% CI, 2.45-13.56; p<0.0001, *I^2^*=0%. *APC* promoter more frequently hypermethylated in CRC stage I compared to normal colorectal tissue, OR was 13.42, 95% CI, 3.66-49.20; p<0.0001, *I^2^*=31%. The risk of incidence of CRC was significantly correlated to *APC* promoter hypermethylation, pooled OR was 9.80, 95%CI, 6.07-15.81; p<0.00001, *I^2^*=43%. *APC* methylation was not associated with grade, stage of CRC as well as tumor location, patients’ gender, and smoking behavior. The results indicate that *APC* promoter hypermethylation is an early event in carcinogenesis of CRC, could be a valuable diagnostic marker for early-stage CRC. *APC* methylation is not significantly associated with overall survival in patients with CRC. APC is a potential drug target for development of personalized treatment.

## INTRODUCTION

Colorectal cancer (CRC) is one of the most common types of cancer worldwide and results from the accumulation of genetic and epigenetic alterations in colonic mucosa cells, which ultimately leads to colorectal adenoma, advanced to invasive and metastatic CRC. Unfortunately, the prognosis of CRC in late stages is still poor and the search of novel diagnostic and prognostic biomarkers is highly desired to prevent CRC-related mortality. During last decade, epigenetic alterations have been reported to play an important role in many cancers initiation, progression, and metastasis [1, 2]. DNA methylation within CpG island in promoter region of genes is associated with the loss of gene expression and is observed in many types of cancers including CRC. *Adenomatous polyposis coli* (*APC*), a suppressor gene, is located at chromosomal band 5q21-q22 and consists of 15 exons. *APC* was discovered by genetic linkage analysis in familial adenomatous polyposis (FAP) and was reported by Kinzler [3], Nishisho [4], Joslyn [5] and Groden [6]. Recently APC is thought of as a negative regulator in Wnt/beta-catenin signaling pathway. Loss of APC function leads to the destabilization and degradation of beta-catenin, and the nuclear accumulation of beta-catenin results in the activation of T-cell factor/LEF target gene and initiates tumorgenesis [7, 8]. *APC* along with several other inactivated genes plays a prognostic indicatory role in squamous cell and adenocarcinoma of esophagus, bladder and lung cancers [9]. In the past two decades, *APC* promoter hypermethylation was frequently observed in sporadic and familial CRC. However, the association between clinicopathological significance and *APC* methylation was under investigated. The present article aims to summarize the most recent findings concerning the use of epigenetic (mainly related to DNA methylation) biomarkers for CRC diagnosis, progression, and response to treatment.

## RESULTS

### Identification of relevant studies

**Figure 1:**
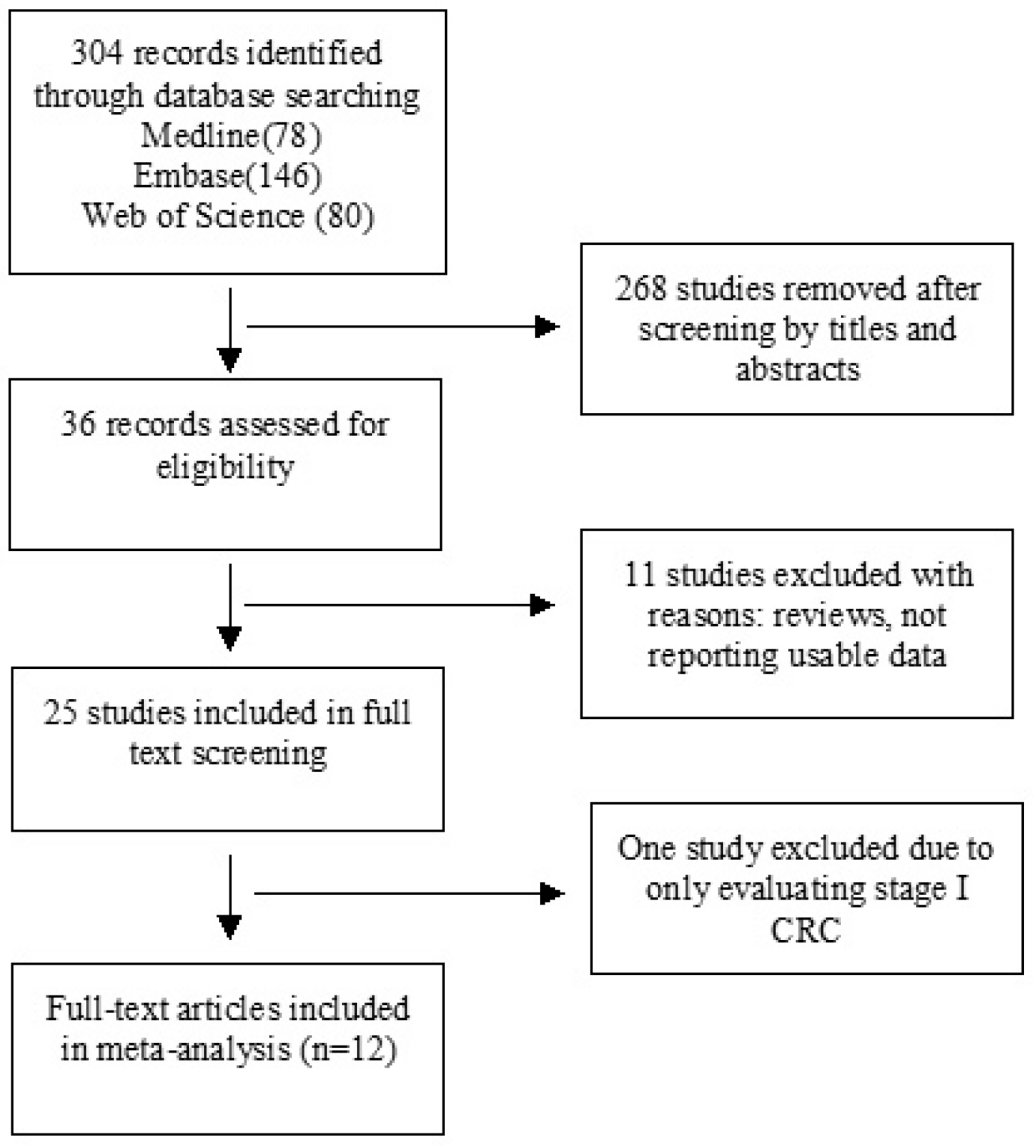
Schematic flow diagram for selection of included studies

36 publications were identified by the search method as described above. Eleven of those were excluded due to laboratory studies, non-original articles (review), or studies irrelevant to the current analysis. Eventually, there were 24 studies included in the final meta-analysis as shown in Figure 1.

### Study characteristics

24 studies published from 2004 to 2015 were eligible for meta-analysis. A total of 1396 samples including CRC, colorectal adenoma and normal control tissues from Greece, Iran, Sweden, Vietnam, China, South Korea, Japan, UK, Kashmir, Czech Republic, Australia, Netherland, Germany, Norway, and USA were included in the analysis. Their basic characteristics are summarized in Table 1.

**Table 1:** Main characteristics of included studies

Author	Year	Country	Methods	Histology			Tumor location		Stage (TNM)		Grade		Smoking status	
				NCT	Ade	CRC	Proximal	Distal	I+II	III+IV	L	H	+	-
Michailidi [33]	2015	Greece	MSP	12/14	-	18/61	-	-	-	-	-	-	-	-
Samaei [34]	2014	Iran	MSP	0/125	-	44/125	29/36	18/50	24/56	22/69	34/100	10/25	-	-
Dimberg [35]	2013	Sweden/Vietnam	MSP	66/101	-	50/101	-	-	-	-	-	-	-	-
Pack [36]	2013	Korea	MSP	2/10	6/10	-	-	-	-	-	-	-	-	-
Qiu [37]	2014	China	MSP	1/10	45/67	44/70	21/35	23/35	-	-	-	-	-	-
Gay [38]	2012	UK	Pyrosequencing	-	-	-	22/59	48/112	22/87	14/69	61/141	6/25	-	-
Kang [39]	2012	Korea	Q-MSP	2/14	-	52/100	-	-	28/52	24/48	-	-	12/20	40/80
Leong [40]	2011	UK	Q-MSP	1/19		22/51							-	-
Naghibalhossaini [41]	2011	Iran	MSP	-	-	-	30/30	73/80	66/71	26/28	48/49	42/48	53/56	50/54
Sameer [42]	2011	Kashmir	MSP	-	-	-	-	-	-	-	9/38	38/48	33/55	14/31
Vasovcak [43]	2011	Czech Republic	MS-MLPA	-	-	-	26/34	45/60	38/56	30/44	-	-	-	-
Belshaw [44]	2010	UK	Q-MSP	2/8	-	2/5	-	-	-	-	-	-	-	-
Kim [45]	2010	Korea	Pyrosequencing	-	-	-	9/68	36/217	28/169	24/116	40/253	5/32	-	-
Kamiyama [46]	2009	Japan	Q-MSP	-	-	-	6/20	10/25	-	-	-	-	-	-
Derks [47]	2006	European/USA	MSP	3/18	17/34	10/18			-	-	-	-	-	-
Iacopetta [27]	2006	Australia	Q-MSP	-	-	-	24/90	33/106	-	-	41/122	6/28	-	-
Brandes [48]	2005	Netherlands	MSP	-	-	-	-	-	19/30	6/14	-	-	-	-
Chen [49]	2005	Germany	MSP	0/14	-	17/34	-	-	-	-	-	-	-	-
Ebert [50]	2005	Germany	MSP	0/21		10/47	-	-	-	-	-	-	-	-
Kim [51]	2005	Korea	MSP	6/40		7/36	-	-	-	-	-	-	-	-
Bai [52]	2004	China	MSP	1/34		28/47	-	-	-	-	-	-	-	-
Lee [53]	2004	Korea	MSP	3/24	34/95	76/149	29/56	47/93	-	-	-	-	-	-
Lind [54]	2004	Norway	MSP	-	-	-	7/18	13/37	13/31	8/26	16/44	4/11	-	-
Xu [55]	2004	China	MSP	0/6	2/8	5/65	-	-	-	-	-	-	-	-

MSP: methylation-specific PCR; MS-MLPA: methylation specific multiplex ligation-dependent probe amplification, NCT: normal control tissue; Ade: adenoma; CRC: colorectal carcinoma; L: low grade; H: high grade.

### The correlation of *APC* hypermethylation with clinicopathological features

1. The inactivation of *APC* through promoter hypermethylation in adenoma and CRC.

**Figure 2:**
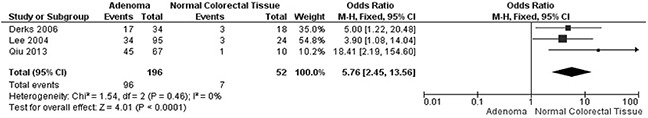
Forest plot for *APC* methylation in adenoma and normal colorectal tissue

**Figure 3:**
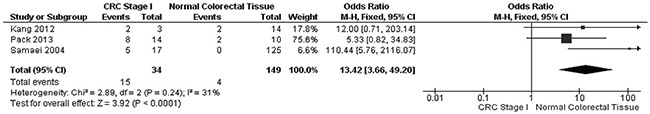
Forest plot for *APC* methylation in CRC stage I and normal colorectal tissue

**Figure 4:**
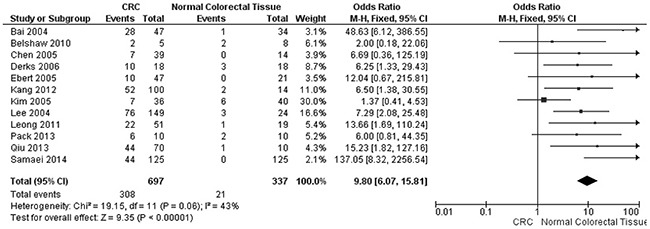
Forest plot for *APC* methylation in CRC and normal colorectal tissue

*APC* promoter hypermethylation was an early event in carcinogenesis. The frequency of *APC* promoter hypermethylation was significantly increased in adenoma than in normal colorectal tissues, OR was 5.76, 95%CI, 2.45-13.56; p<0.0001, *I^2^*=0% (Figure 2). *APC* promoter was more frequently hypermethylated in CRC stage I than normal colorectal tissue, OR was 13.42, 95% CI, 3.66-49.20; p<0.0001, *I^2^*=31% (Figure 3). The risk of incidence of CRC was significantly correlated to *APC* promoter hypermethylation, pooled OR was 9.80, 95%CI, 6.07-15.81; p<0.00001, *I^2^*=43% (Figure 4).

2. *APC* promoter hypermethylation was not associated with grade and stage of CRC.

**Figure 5:**
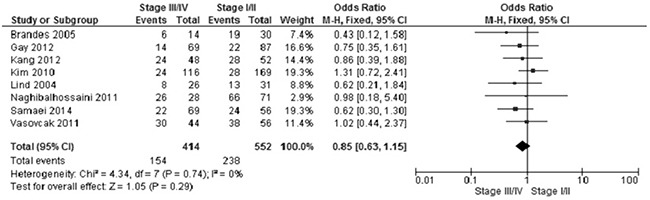
Forest plot for *APC* methylation in stage III/IV and stage I/II of CRC

**Figure 6:**
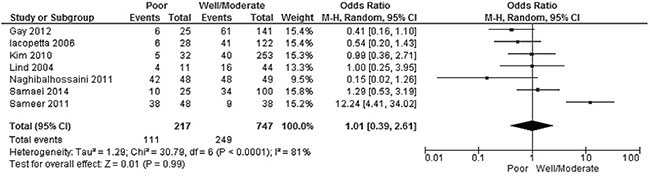
Forest plot for *APC* methylation in high grade and low grade of CRC

The frequency of *APC* promoter hypermethylation was similar between low and high grade of CRC, pooled OR was 1.01, 95%CI, 039-2.61; p=0.99, *I^2^*=81% (Figure 5). There was no difference when comparing the frequency of *APC* promoter hypermethylation between I/II stage and III/IV stage of CRC, pooled OR was 0.85, 95%CI, 0.63-1.15; p=0.29, *I^2^*=0% (Figure 6).

3. There was no statistically significant association between *APC* methylation status and other clinical parameters, including tumor location, gender and smoking status of CRC patients.

**Figure 7:**
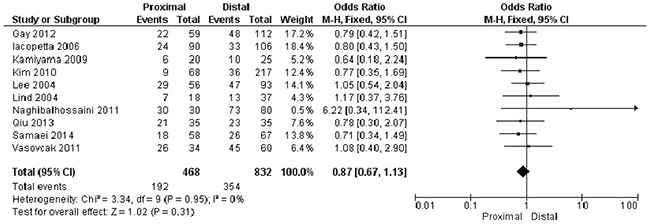
Forest plot for the correlation of *APC* hypermethylation and location of CRC

Proximal versus distal: OR was 0.87, 95%CI, 0.67-1.13, p=0.31, *I^2^*=0% (Figure 7).

**Figure 8:**
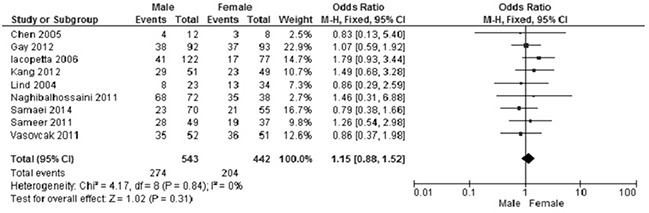
The frequency of *APC* hypermethylation was similar in male and female CRC patients

Male versus female: OR was 1.55, 95%CI, 0.88-1.52, p=0.31, *I^2^*=0% (Figure 8).

**Figure 9:**
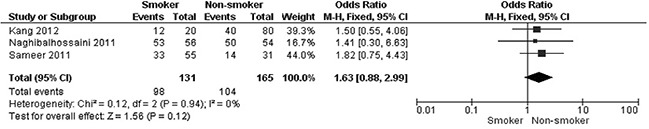
Plot for the relationship of *APC* hypermethylation and smoking status of patients with CRC

Smoker versus non-smoker: OR was 1.63, 95%CI, 0.88-2.99, p=0.12, *I^2^*=0% (Figure 9).

**Figure 10:**
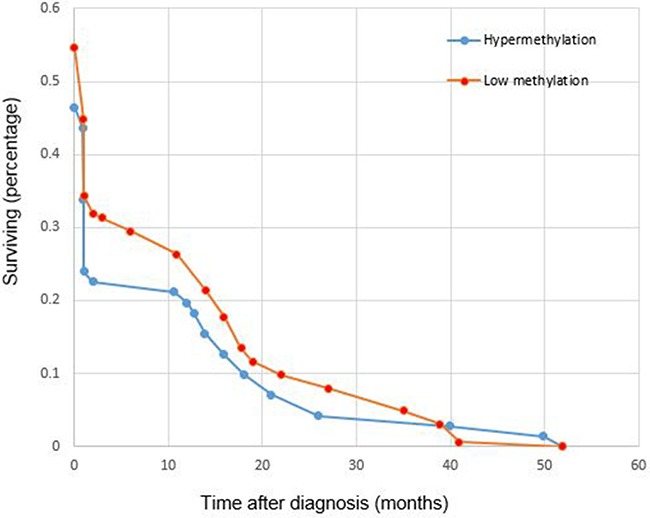
Plot for the overall survival of CRC patient with different *APC* promoter methylation status Blue circles represent cases with *APC* hypermethylation, red circles represent cases with *APC* low methylation. Test p-value 0.5798.

4. Overall survival was analyzed by selecting Colorectal Adenocarcinoma (TCGA, Nature 2012) [10] and gene *APC* via cBioPortal for provisional data. The survival curve was plotted on 236 cases (methylation HM27) which included 72 cases with *APC* hypermethylation (methylation beta-value was more than 0.3) and 164 cases with *APC* low methylation (methylation beta-value was more than 0.3). T-test p-value was 0.5798, indicating *APC* methylation was not significantly associated with overall survival in patients with CRC (Figure 10).

### Sensitivity analyses and publication bias

**Figure 11:**
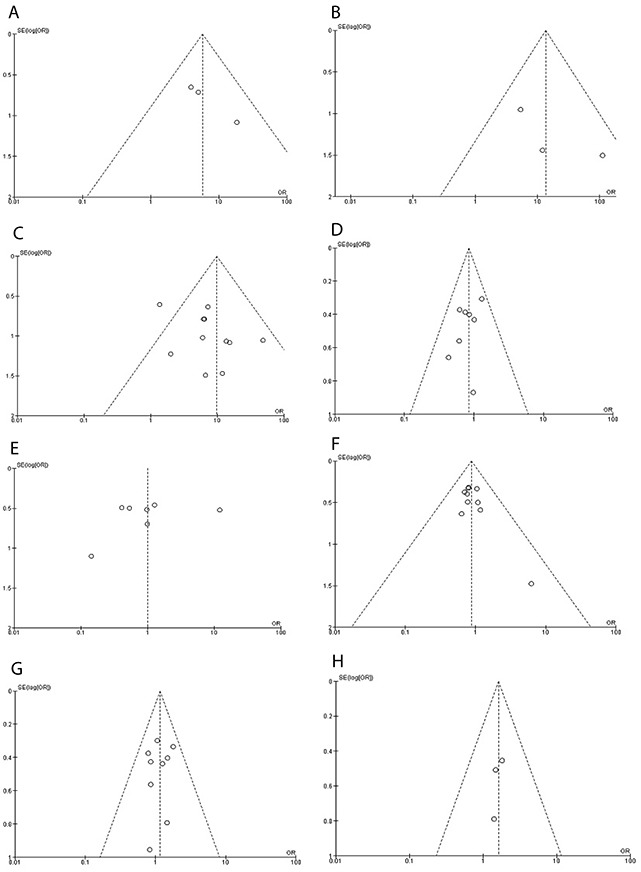
Funnel plot for publication bias **A**. *APC* methylation in adenoma and normal colorectal tissue. **B**. *APC* methylation in CRC stage I and normal colorectal tissue. **C**. *APC* methylation in CRC and normal colorectal tissue. **D**. *APC* methylation in stage III/IV and stage I/II of CRC. **E**. *APC* methylation in high grade and low grade of CRC. **F**. The correlation of *APC* hypermethylation and location of CRC. **G**. *APC* hypermethylation in male and female CRC patients. **H**. The relationship of *APC* hypermethylation and smoking status of patients with CRC. S.E.: standard error; area of the circle represents the weight of individual study.

To minimize the effect of confounders, a sensitivity analysis, in which one study was removed at a time, was conducted to assess the result stability. The pooled ORs were not significantly changed, indicating the stability of our analyses. The funnel plots demonstrates no obvious asymmetry (Figure 11A-11H), suggesting the absence of publication biases in the meta-analysis of *APC* hypermethylation and clinicopathological features.

## DISCUSSION

CRC is thought to develop from adenomatous polyps following the accumulation of mutations which includes the activation of oncogenes and the inactivation of tumor suppressor genes [11–15]. Tumor suppressor genes associated with CRC include *APC*, *p53*, *BRAF* and *DCC* [10, 15], and the loss of APC has been observed in approximately 70-80% of CRC [16–19], but the association between *APC* promoter methylation and clinicopathological significance in CRC is unclear. The current study systematically reviewed all published evidence before July 2016 and synthesized data from three studies and 196 adenoma samples using a meta-analysis. Our result indicated that the *APC* promoter is 5.76 times more frequently hypermethylated in adenoma than in normal colorectal tissue. In addition, the frequency of *APC* hypermethylation in CRC stage I was 13.42 times higher than in normal colorectal tissue. Patients with early-stage CRC could expect a long survival with surgery alone, but about 50% stage III and 25% stage II will relapse and need adjuvant chemotherapy [20]. Therefore, *APC* hypermethylation as a valuable early diagnostic marker could contribute to making the decision whether or not to accomplish chemotherapy. Ding et al published a meta-analysis of the association between *APC* promoter methylation and colorectal cancer, in which the *APC* promoter hypermethylation has not been analyzed in early-stage CRC.

We pooled 12 studies and included 762 CRC samples and 343 normal colorectal tissues, and the data demonstrated the frequency of *APC* hypermethylation in CRC was 9.8 times higher than in normal colorectal tissues; the heterogeneity was 43%. We removed three studies that caused higher heterogeneity (the heterogeneity was 83%, OR was 4.35, 95% CI was 1.56-12.12). One of the three studies (Xu et al.) reported low frequency of *APC* methylation in CRC (7.7%) compared to other studies (*APC* methylation rate arranged from 17.9% to 62.8%), two studies (Dimberg at al. and Michailidi et al.) reported high rate of *APC* methylation (65.3% and 85.7%) in normal colorectal tissues compared to other studies (*APC* methylation rate arranged 0-25%). The frequency of *APC* hypermethylation was similar between CRC and adenoma (data not shown), this result is consistent with previous study [21]. Our results suggest that *APC* promoter hypermethylation is an early event during colorectal carcinogenesis. Previous evidence suggest that *APC* methylation is not a “second hit” in two hit model of *APC* mutation in tumor [22]. This explains why *APC* promoter hypermethylation was an early event during the development of CRC. As the changes in *APC* promoter hypermethylation are reversible, demethylation with drug could delay carcinogenesis and progression of CRC. Previous studies showed that adenoma formation in *APC* ^min/+^ was inhibited by 5-aza-deoxycitidine, a demethylation agent [23]. In addition, Eads et al demonstrated that the expression of full-length *Dnmt3b1* enhanced the number of colon tumors in *APC* min/+ mice by approximately twofold and increased the average size of colonic microadenomas [24, 25]. Taken together, APC is a potential drug target for the development of personalized therapy in patients with CRC; further investigation is required in future.

Among the included studies, the frequency of *APC* methylation have varied greatly from 17.9% to 62.8%. This phenomenon maybe due to ethnic differences, different PCR primers used in the detection, as well as cancer heterogeneity. According to recent classification system, CRC is classified into two major groups: 1) hypermutated cancers with either microsatellite instability due to defective mismatch repair or ultramutated cancers with DNA polymerase epsilon proofreading mutations; 2) non-hypermutated, microsatellite stable cancers with a high frequency of DNA somatic copy number alterations, which showed common mutations in *APC, TP53, KRAS, SMAD4* and *PIK3CA*. *APC* methylation is present more often in the first group of tumors with microsatellite-stable compared to the second group with microsatellite instability [26–29]. In addition, the *APC* methylation was inversely associated with *TIMP3, TP53* and *BRAF* methylation [27]. Previous publications reported that *APC* was less frequently mutated alone, more commonly mutated with KRAS, TP53, PIK3CA and SMAD [22, 27], suggesting that the *APC* mutation occurs early in carcinogenesis, the alterations of other genes were involved during the transition from adenoma to carcinoma. *APC* methylation combined with the mutation of other genes could be a valuable biomarker for diagnosis and prognosis of CRC. Further study is necessary to substantiate this issue.

We pooled seven studies and included 964 samples and analyzed the relationship of *APC* promoter methylation with the grade of CRC; the power was 0.89, which indicated that *APC* promoter hypermethylation is not associated with grade. Furthermore, present analysis showed that *APC* promoter hypermethylation is not correlated with stages of CRC, since the power of the study is small, further study with a larger number of samples is need to confirm this relationship.

Consistent results were shown in sensitivity analyses, indicating the stability of our analyses. All funnel plots did not show any obvious asymmetry, suggesting there is no publication bias in the meta-analysis. This study has several potential limitations. First, selection bias may exist since only publications in English and Chinese were included in the present study, which could affect the accuracy of results in certain extent. Caution should be taken when our findings are interpreted. Second, the possibility of information, selection biases and unidentified confounders could not be completely excluded because all of the included studies were observational. Third, our results showed that there is no significant correlation between *APC* methylation and gender, smoking behavior of CRC patients as well as tumor locations; since the power of the study is small, further evaluation with a larger number of samples is required in future.

In summary, our meta-analysis indicates that *APC* promoter hypermethylation is an early event of carcinogenesis of CRC, and *APC* methylation combined with the mutation of other genes could be a valuable biomarker for diagnosis and prognosis of CRC. *APC* promoter methylation is not significantly associated with overall survival in patients with CRC. APC is a potential drug target for development of personalized therapy.

## METHODS

### Search strategy

We performed this meta-analysis in accordance with the Preferred Reporting Items for Systematic Reviews and Meta-analysis (PRISMA) statement [30]. We searched the database of Medline, Web of science, and Embase up to July, 2016 without language limitations. The following items were used for searching: APC, adenomatous polyposis coli, methylation, neoplasm, tumor, colorectal carcinoma, and CRC. A manual search using references from retrieved articles was performed for additional pertinent studies. We chose the most complete study to avoid duplication when the same populations were reported in several publications.

### Study selection

Studies were included if they met the following inclusion criteria: 1) investigation *APC* methylation status and clinicopathological significance in CRC. 2) case-control, and cohort studies published as original studies. 3) studies that provided sufficient data to calculate ORs and 95% confidence interval (CI).

Exclusion criteria were: 1) lack of sufficient data on *APC* methylation and clinicopathological features in CRC, 2) reviews, case report, conference abstract and expert opinion and letters, 3) all publications regarding in vitro studies.

### Quality assessment

The quality of each study was individually evaluated by each investigator utilizing Newcastle-Ottawa quality assessment scale [31]. All observational studies were considered moderate to high quality, with median Newcastle-Ottawa quality assessment scale of 7 (range, 6-9) (data not shown).

### Data extraction

A standardized data extraction form was used. Eligible studies were reviewed and the following data were extracted: (1) first author's name, (2) year of study, (3) study location, (4) methylation detect methods, (5) sample size, (5) tumor location, cancer TMN stages and grade (6) gender and smoking status of participants.

### Survival analysis with TCGA data

Overall survival was analyzed by selecting Colorectal Adenocarcinoma (TCGA, Nature 2012) and gene *APC* via cBioPortal for provisional data. *APC* methylation and overall survival data were downloaded. Hypermethylation and Low methylation were sorted out according to methylation beta-value. If the methylation beta-value was more than 0.3, the case was considered as hypermethylation, if the methylation beta-value was less than 0.3, the case was considered as low methylation. The overall survival was plotted on 236 cases (methylation HM27) which included 72 cases with *APC* hypermethylation (methylation beta-value was more than 0.3) and 164 cases with *APC* low methylation (methylation beta-value was less than 0.3) with Excel 2013.

### Statistical analysis

Review Manage 5.3 from the Cochrane Collaboration was used for data analysis. Odds Ratio with 95% confidence intervals (CIs). This statistic was complemented with the *I^2^* statistic, which quantifies the proportion of the cumulative variation across studies that is due to heterogeneity rather than chance. When heterogeneity was not an issue (*I^2^* values <50%), a fixed effect model was used to calculate parameters. When there was substantial heterogeneity (*I^2^* values ≥50%), a random-effects model was used to pool data and attempt to identify potential sources of heterogeneity based on subgroup analyses. Two sided statistical tests and p-value were used.

### Evaluation for publication bias

The presence of publication bias was assessed by funnel plots of logarithm of odds ratios versus their standard errors [32].
